# Moxibustion against Cyclophosphamide-Induced Premature Ovarian Failure in Rats through Inhibiting NLRP3-/Caspase-1-/GSDMD-Dependent Pyroptosis

**DOI:** 10.1155/2021/8874757

**Published:** 2021-02-04

**Authors:** Cai-rong Zhang, Wei-na Zhu, Wei Tao, Wan-qi Lin, Chang-cheng Cheng, Han Deng, Guang-xia Ni

**Affiliations:** ^1^Department of Acupuncture and Moxibustion, Nanjing Hospital of Chinese Medicine affiliated to Nanjing University of Chinese Medicine, 157 Daming Road, Nanjing, China; ^2^Department of Central Laboratory, Nanjing Hospital of Chinese Medicine Affiliated to Nanjing University of Chinese Medicine, 157 Daming Road, Nanjing, China; ^3^College of Acupuncture and Massage, College of Regimen and Rehabilitation, Nanjing University of Chinese Medicine, 138 Xianlin Avenue, Nanjing, China

## Abstract

Premature ovarian failure (POF) is a clinical term used to describe a condition in which women present with amenorrhoea, hypergonadotropic hypogonadism, and infertility under 40 years old, which are mainly characterized by ovarian granulosa cell inflammation and death. Pyroptosis is a proinflammatory form of programmed cell death. However, the roles of pyroptosis in POF and moxibustion (Mox) on pyroptosis in POF have not been elucidated. The aim of the present study was to investigate the protective effect of moxibustion against cyclophosphamide- (CP-) induced POF and to determine the underlying mechanisms. The results indicated that Mox could decrease the follicle-stimulating hormone (FSH) and luteotropic hormone (LH) and increase estradiol (E2) in serum, which indicated that it could improve ovarian reserve capacity. Mox also ameliorated CP-induced ovarian injury accompanied by decreased levels of interleukin-1*β* (IL-1*β*), IL-18, and gasdermin D (GSDMD), which are key features of pyroptosis. Further investigation showed that Mox alleviated POF through NLRP3-mediated pyroptosis. On the one hand, Mox directly inhibited TXNIP/NLRP3/caspase-1 signaling-induced pyroptosis, and on the other hand, it indirectly decreased NLRP3, pro-IL-1*β,* and pro-IL-18 through inhibiting TLR4/MyD88/NF-*κ*B signaling. Our results show that Mox might be a new therapeutic strategy for the treatment of POF.

## 1. Introduction

Premature ovarian failure (POF) is a clinical term used to describe a condition in which women present with amenorrhoea, hypergonadotropic hypogonadism, and infertility under 40 years old. The incidence of POF in women under 40 and 30 years of age has been reported to be 1% and 0.1%, respectively [1]. POF is usually characterized by low gonadal hormone (estrogen) levels and high gonadotropins (LH and FSH) levels [2, 3]. Secondary amenorrhoea, which is usually based on POF, makes the patient suffer from the psychological distress associated with fertility loss. In addition, untreated ovarian failure has an increased risk of developing osteoporosis, cardiovascular disease, and cognitive decline [4, 5]. Thus, optimizing ovarian function is important for women with POF. The main mechanism of POF is follicle depletion and dysfunction, manifested by ovarian atrophy and cortical fibrosis [6]. During follicular development, a large number of follicles undergo atresia, a process tightly controlled by the fine balance between survival and death factors [7]. Therefore, it is important to determine the molecular mechanisms of ovarian injury in POF.

Cyclophosphamide (CP), an alkylating molecule, is used in numerous cancer chemotherapeutic regimens. Besides the beneficial effect of CP on target areas of the tumor, it could also damage various organs depending on the age and sex of patients [8]. In clinical work, female patients with cancer who were treated with CP can experience infertility due to premature ovarian failure [9]. Chemotherapy induces apoptosis of growing follicles and fibrosis ovaries in the stromal blood vessels [10]. It has a damaging effect on ovarian tissue and increases the risk of POF. Therefore, CP was used in the present study to induce POF in female rats. Ovarian failure is accompanied by a chronic inflammatory response [11]. Pyroptosis is a form of programmed cell death, and the activation of pyroptosis is closely related to inflammation processes [12]. The features of pyroptosis are gasdermin family-mediated pore formation on the plasma membrane, cell swelling, and plasma membrane disruption, as well as release of proinflammatory intracellular contents including IL-1*β* and interleukin-18 (IL-18). Gasdermin D (GSDMD) is cleaved by activated caspase-1 to trigger pyroptosis by forming membrane pores [13]. Activation of inflammasomes, especially NLR family pyrin domain containing 3 (NLRP3), plays an important role in pyroptosis. NLRP3 promotes caspase-1-mediated IL-1*β*, IL-18, and cleaved GSDMD. The molecular mechanisms of inflammasomes and pyroptosis are as follows. Firstly, the transcription factor promotes the production of proinflammatory factors such as pro-IL-1*β*, pro-IL-18, NLRP3, and caspase-1. This is mainly regulated by nuclear factor-*κ*B (NF-*κ*B) [14]. Secondly, it activates the inflammatory complex, including NLRP3, ASC, and procaspase-1. This is mainly regulated by Thioredoxin Interacting Protein (TXNIP) [15]. Long-term chronic inflammatory response, including IL-1*β*, could induce ovarian cell damages. Therefore, we hypothesized that pyroptosis may play an important role in the progress of ovarian failure.

Moxibustion (Mox) is a traditional Chinese therapy using burned moxa stick made from dried mugwort. The combustion of the mugwort permits transmission of heat to the body that has various pathologic changes [16]. Previous studies showed that Mox has beneficial effects on arthritis, knee osteoarthritis, and pain [17, 18]. It indicated that such pleiotropic effects include improving immune function and inhibiting oxidative stress and apoptosis. Evidence suggests that Mox has a good effect on POF [19], but the underlying mechanism still remains unclear. The present study was designed to explore the effect of moxibustion on POF and its underlying mechanism.

## 2. Materials and Methods

### 2.1. Reagents and Kits

Moxa sticks were provided by Nanjing Hospital of Traditional Chinese Medicine (Nanjing, China). Cyclophosphamide was purchased from Jiangsu Sheng Di Pharmaceutical Co., Ltd. (Nanjing, China). Enzyme-linked immunosorbent assay (ELISA) kits of follicle-stimulating hormone (FSH), estradiol (E2), luteotropic hormone (LH), interleukin-1*β* (IL-1*β*), and IL-18 were obtained from Elabscience (Wuhan, China). Primary antibodies against TXNIP, MyD88, NF-*κ*B, ASC, cleaved caspase-1, Gasdermin D, and GAPDH were produced by Cell Signaling Technology (USA). NLRP3, p-NF-*κ*B, IL-1*β*, and IL-18 were produced by Abcam (UK). TLR4 was produced by Santa Cruz Biotechnology (USA). The critical chemicals and antibodies were listed in Supplementary Table 1.

### 2.2. Animals

Twelve-week-old female Sprague Dawley (SD) rats were purchased from the Shanghai SLAC Laboratory Animal Co. Ltd. All animals were housed under specific pathogen-free (SPF) conditions (24 ± 2°C room temperature, 65 ± 5% humidity, and 12/12 h light-dark cycles) with drinking water and food available *ad libitum*. The animal welfare and experimental procedures adhered strictly to the dictates of the National Institutes of Health (NIH) Guide for the Care and Use of Laboratory Animals.

### 2.3. Experimental Procedure

**Figure 1:**
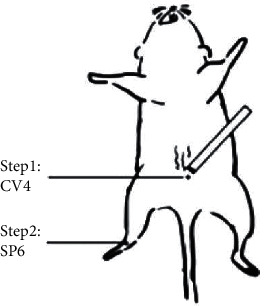
Moxibustion at Guanyuan (CV4) and Sanyinjiao (SP6) acupoints in rats.

After accommodation for one week, rats were randomly allocated into three groups (*n* = 15 for each group): (1) control group, (2) model group (cyclophosphamide), and (3) moxibustion (Mox) group. The rats of the model and Mox groups were intraperitoneally injected with 50 mg/kg of CP on the first day and then 8 mg/kg for 15 consecutive days, while the control group was injected with 0.9% saline instead of CP. Moxibustion with an ignited moxa stick was administered to rats at Guan-yuan (CV4, located on the midline 3 cm below the umbilicus) and Sanyinjiao (SP6, located on the inside of the leg just above the ankle) acupoints, respectively (as shown in Figure 1). Each acupoint was given moxibustion for 10 min twice per day for 3 weeks. All rats were sacrificed under anesthesia and blood and ovarian were collected for further study.

### 2.4. Histological Analysis

Ovarian samples were excised immediately, fixed in 4% paraformaldehyde (PFA), embedded in paraffin wax, and cut into slices of 4 *μ*m thicknesses. The paraffin sections were dewaxed in xylene, dehydrated in ethanol, and stained with hematoxylin-eosin (HE), and then they were observed under light microscopy at 100x magnifications.

### 2.5. Enzyme-Linked Immunosorbent Assay (ELISA)

The blood was centrifuged at 3000 rpm for 10 min at 4°C to obtain serum. The levels of hormone secretion (FSH, E2, and LH) and inflammatory cytokines (IL-1*β* and IL-18) in serum were determined by ELISA kits according to the manufacturer's instructions (Elabscience, China).

### 2.6. Immunohistochemical Staining

The proteins of TLR4 and p-NF-*κ*B in ovarian were evaluated by immunohistochemical staining. Briefly, the ovary was fixed with 4% PFA, embedded in paraffin, and cut into slices of 4 *μ*m thicknesses. The paraffin sections were dewaxed in xylene, dehydrated in ethanol, and microwaved in sodium citrate buffer for 20 min to repair the antigen. The samples were incubated with 3% hydrogen peroxide for 30 min to block endogenous peroxidase and blocked with 5% goat serum for 30 min. All samples were treated with primary antibodies TLR4 (1 : 50) and p-NF-*κ*B (1 : 200) at 4°C overnight. The next day, each sample was incubated with goat anti-rabbit/mouse IgG for 30 min and then horseradish enzyme tag chain enzyme avidin for 30 min. Then, samples were stained with 3-3′diaminobenzidine (DAB) and restained with hematoxylin. The samples were observed under light microscopy at 100x magnifications.

### 2.7. Western Blotting

Total protein of rat ovarian tissues was extracted for western blotting. The samples were minced and homogenized in ice-cold RIPA buffer containing 1 mM PMSF, protease, and phosphatase inhibitor (Beyotime Biotechnology, China). Then, the samples were centrifuged at 12000 *g*, 4°C for 15 min, supernatants were collected, and the protein concentration was measured by the BCA assay kit (Beyotime Biotechnology, China). The proteins were subjected to 10% SDS-PAGE electrophoresis, transferred to polyvinylidene fluoride membranes (PVDF), and then blocked with 5% skim dried milk for 2 h. Primary antibodies, including TLR4 (1 : 200), MyD88 (1 : 1000), NLRP3 (1 : 1000), ASC (1 : 1000), cleaved caspase-1 (1 : 1000), p-NF-*κ*Bp65 (1 : 1000), NF-*κ*Bp65 (1 : 1000), IL-1*β* (1 : 1000), IL-18 (1 : 1000), and GAPDH (1 : 1000), were incubated overnight at 4°C. After that, they were incubated with HRP-conjugated secondary antibodies for 2 h. The samples were visualized by an enhanced chemiluminescence (ECL) advanced kit and a gel imaging system.

### 2.8. Statistical Analysis

All data were presented as mean ± SD (standard deviation). Normality was checked for all data before comparisons using one-way ANOVA followed by Tukey's multiple comparison test. A *p* value less than 0.05 was considered statistically significant.

## 3. Results

### 3.1. Mox Could Alleviate CP-Induced Ovarian Damage and Abnormal Hormone Secretion

**Figure 2:**
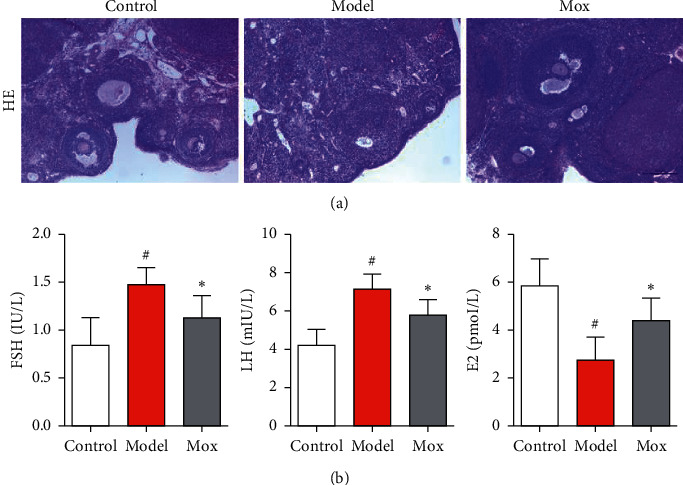
Mox could alleviate CP-induced ovarian damage and abnormal hormone secretion. (a) Mox could alleviate CP-induced ovarian damage by HE staining. The photomicrographs for HE staining of ovarian tissue sections. (b) Mox inhibited CP-induced increase of FSH and LH and decreased E2 in serum determined by ELISA kits (*n* = 8). All the data were presented as mean ± SD. Compared with the control group, #*p* < 0.05; compared with the model group, *∗p* < 0.05.

Hematoxylineosin staining was used to analyze the histological changes of CP-induced ovarian damage. As shown in Figure 2(a), the texture of ovarian tissues, including primordial follicles, antral follicles, and cumulus oophorus, was intact in the control group. The model group showed increased ovarian atrophy and stroma empty space, and ovarian granulosa cell damage was found compared with the control group. However, compared with the model group, the above pathological damage was alleviated by Mox treatment. As is well known, POF could induce abnormal hormone secretion. We used to detect the levels of serum hormone secretion by ELISA kit. As shown in Figure 2(b), the concentrations of FSH and LH were increased, and E2 was decreased in the serum of the model group. As expected, Mox decreased the concentration of FSH and LH and increased E2 in serum. The above studies indicate that Mox could alleviate CP-induced POF. However, the mechanisms of Mox are not as well understood.

### 3.2. Mox Could Alleviate CP-Induced Ovarian Pyroptosis

**Figure 3:**
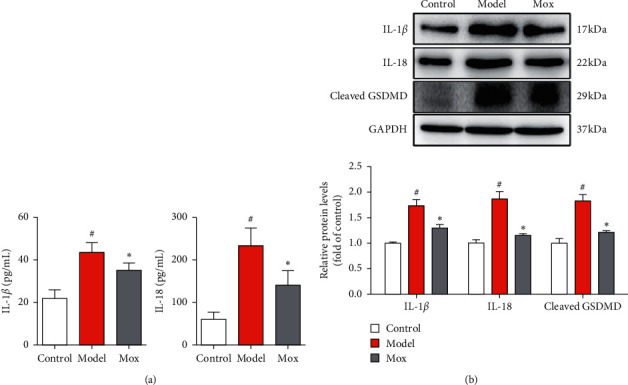
Mox could alleviate CP-induced ovarian pyroptosis. (a) Mox could inhibit the CP-induced increase of IL-1*β* and IL-18 in serum determined by ELISA kits (*n* = 8). (b) Mox inhibited CP-induced increased protein expression of IL-1*β*, IL-18, and cleaved GSDMD by western blot (*n* = 3). All the data were presented as mean ± SD. Compared with the control group, #*p* < 0.05; compared with the model group, *∗p* < 0.05.

In order to study whether pyroptosis occurred in CP-induced POF rats, we further detected the pyroptosis markers, including IL-1*β* and IL-18, through ELISA kits. As expected, IL-1*β* and IL-18 were increased in POF rats (Figure 3(a)). Mox could decrease the IL-1*β* and IL-18 in POF rats. Gasdermin D (GSDMD), which is a pore-forming protein that promotes the secretion of IL-1*β* and IL-18, could induce pyroptosis. The proteins of IL-1*β*, IL-18, and cleaved GSDMD were significantly increased in POF rats, which further indicated that pyroptosis occurred in the POF rats. Meanwhile, Mox decreased the proteins of IL-1*β*, IL-18, and cleaved GSDMD in POF rats (Figure 3(b)). Preliminary studies showed that pyroptosis occurred in the POF rats, and Mox could inhibit pyroptosis.

### 3.3. Mox Inhibited Pyroptosis through Inhibiting the TXNIP/NLRP3/Caspase-1 Signaling Pathway

**Figure 4:**
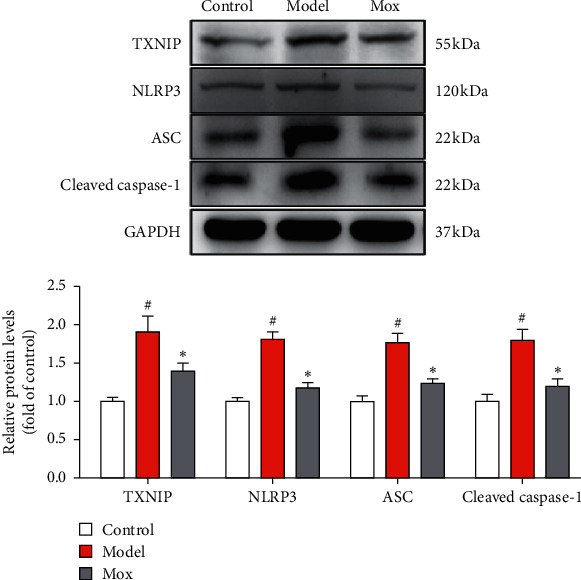
Mox inhibited pyroptosis through inhibiting the TXNIP/NLRP3/caspase-1 signaling pathway. Mox inhibited CP-induced increased protein expression of TXNIP, NLRP3, ASC, and cleaved caspase-1 by western blot (*n* = 3). All the data were presented as mean ± SD. Compared with the control group, #*p* < 0.05; compared with the model group, *∗p* < 0.05.

As is widely known, IL-1*β*, IL-18, and cleaved GSDMD were activated by caspase-1, which was activated by NLRP3. In order to investigate the mechanisms of antipyroptosis in Mox, we detected NLRP3-related protein. As shown in Figure 4, the NLRP3, ASC, and cleaved caspase-1 were remarkably increased in CP-induced POF rats. Mox significantly decreased NLRP3, ASC, and cleaved caspase-1 in POF rats. NLRP3 was always activated by TXNIP. The further study showed that Mox significantly inhibited the CP-induced increase of TXNIP. The above study indicated that TXNIP/NLRP3/caspase-1-medicated pyroptosis plays a key role in POF and Mox suppressed pyroptosis through inhibiting the TXNIP/NLRP3/caspase-1 signaling pathway.

### 3.4. Mox Decreased NLRP3, Pro-IL-1*β*, and Pro-IL-18 through Inhibiting the TLR4/MyD88/NF-*κ*B Signaling Pathway

**Figure 5:**
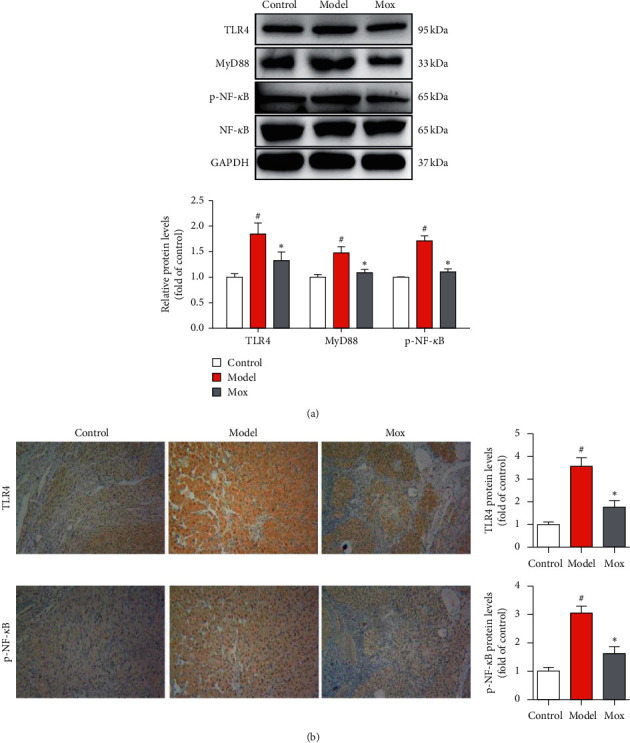
Mox decreased NLRP3, pro-IL-1*β*, and pro-IL-18 through inhibiting the TLR4/MyD88/NF-*κ*B signaling pathway. (a) Mox inhibited CP-induced increased protein expression of TLR4, MyD88, and p-NF-*κ*B by western blot (*n* = 3). (b) Mox inhibited CP-induced increased protein expression of TLR4 and p-NF-*κ*B in the ovarian immunohistochemical staining. Original magnification: 100x. All the data were presented as mean ± SD. Compared with the control group, #*p* < 0.05; compared with the model group, *∗p* < 0.05.

NF-*κ*B-related signaling pathway which mediates the upregulation of pro-IL-1*β*, pro-IL-18, and NLRP3 genes plays the first step of NLRP3 activation, and NLRP3/ASC/caspase-1 could not assemble without NF-*κ*B. In order to investigate whether the NF-*κ*B-related signaling pathway plays an important role in POF, we detected the principal NF-*κ*B-related signaling in the TLR4/MyD88/NF-*κ*B signaling pathway. As expected, the proteins of TLR4, MyD88, and p-NF-*κ*B were significantly augmented in CP-induced POF rats (Figure 5(a)). Mox treatment significantly reversed these elevations induced by CP-induced POF rats (Figure 5(a)). The results were further verified by immunohistochemical (Figure 5(b)). Mox treatment significantly inhibited TLR4 and p-NF-*κ*B in the ovary. Through the above results, we confirmed that Mox decreased NLRP3, pro-IL-1*β*, and pro-IL-18 through inhibiting the TLR4/MyD88/NF-*κ*B signaling pathway and further inhibiting pyroptosis.

## 4. Discussion

**Figure 6:**
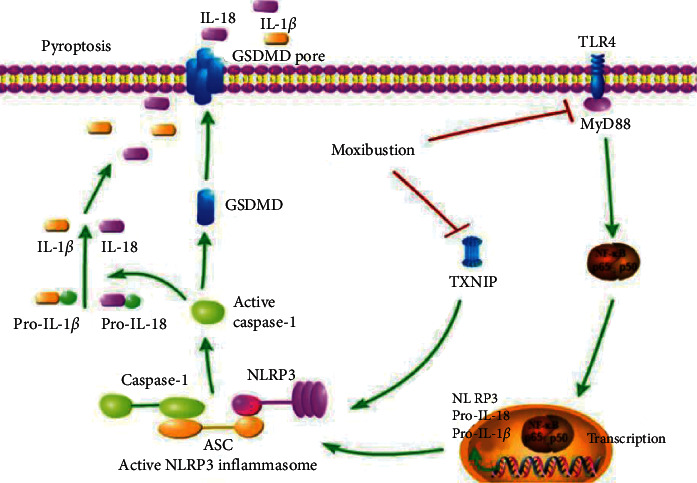
Schematic mechanism of Mox ameliorates POF through inhibiting NLRP3-/caspase-1-/GSDMD-dependent pyroptosis. Mox directly inhibited TXNIP/NLRP3/caspase-1 signaling-induced pyroptosis. Meanwhile, Mox indirectly decreased NLRP3, pro-IL-1*β*, and pro-IL-18 through inhibiting TLR4/MyD88/NF-*κ*B signaling. Our results show that inhibiting pyroptosis or giving Mox might be a new treatment for POF.

Our study showed that Mox could alleviate CP-induced POF through inhibiting pyroptosis and indicated that NF-*κ*B activation and TXNIP/NLRP3 inflammasome triggering caspase-1-dependent pyroptosis play an important role in ovarian injury in rats; the main mechanism is illustrated in Figure 6. The present study provided new insights into the mechanism of moxibustion in treating POF.

CP-induced ovarian damage has been characterized as follows: it decreased various follicles and ovarian mass and increased ovarian atrophy and stroma empty space. Moreover, the secretion of the endocrine hormone is also affected by CP [20]. Mox could significantly reverse the pathological changes of POF induced by CP. In addition, Mox can effectively improve the hormone metabolism as demonstrated by the lowering of FSH and LH levels and increasing the concentration of E2 in serum. These results demonstrated that Mox could suppress the deleterious effects of CP and reverse POF. As a chronic inflammatory disease, POF was affected by numerous cytokines, which initiate and maintain the pathological response in the ovary [21]. Pyroptosis is a proinflammatory form of regulated cell death and plays an important role in tissue homeostasis and immunity. Caspase-1 activation, which subsequently catalyzes the cleavage of the precursor cytokines pro-IL-*β* and pro-IL-18 into a mature and active form, has been considered to be closely related to pyroptosis [22]. Gasdermin D (GSDMD) is a central effector and executor protein of pyroptosis by inducing the formation of large pores in the plasma membrane [23]. We found that the expression of GSDMD and the serum levels of IL-*β* and IL-18 increased in CP-induced rats, while Mox treatment reversed these changes and significantly attenuated ovarian injury.

Nucleotide-binding oligomerization domain receptors (NLRs) are intracellular proteins that participate in mammalian immunity. NLRs can be grouped according to their physiological functions in the immune system. NLRP3 inflammasome complex consists of three submits: NLRP3, apoptosis-associated speck-like protein (ASC), and caspase-1. NLRP3 binds to ASC and then recruits procaspase-1 to form an inflammasome complex. Inflammasome complex cleaves procaspase-1 into caspase-1 and subsequently cleaves pro-IL-1*β* and pro-IL-18 into mature IL-1*β* and IL-18. Caspase-1 also induces GSDMD into cleaved GSDMD and further triggers pyroptosis [24]. Based on our results, Mox could inhibit NLRP3/ASC/caspase-1-mediated pyroptosis. TXNIP is a key antioxidant in the body and is necessary for the activation of the NLRP3 inflammasome via direct interaction with NLRP3. Depending on our results, Mox treatment can significantly inhibit the expression of TXNIP in rats. This led to the speculation that downregulation of TXNIP also plays a role in Mox repression of ovarian injury. Therefore, we hold the opinion that Mox inhibits TXNIP/NLRP3/caspase-1 signaling-medicated pyroptosis and alleviates ovarian failure in POF.

NF-*κ*B-related signaling pathway mediates the upregulation of pro-IL-1*β*, pro-IL-18, and NLRP3 genes and plays the first step of NLRP3 activation, and NLRP3/ASC/caspase-1 could not assemble without NF-*κ*B. The NF-*κ*B family consists of five rel homology-containing proteins, which are normally retained in the cytoplasm through binding to inhibitors of NF-*κ*B (I*κ*B) [25]. Receptor-mediated signaling pathway activates the I*κ*B kinase (IKK) complex and subsequently phosphorylates the inhibitory cytoplasmic I*κ*B*α*, thus allowing NF-*κ*B to translocate the nucleus and to initiate gene transcription. NF-*κ*B-dependent signal is necessary for the activation of NLRP3 [26]. The present study revealed that Mox could inhibit CP-induced activation of NF-*κ*B and NLRP3 in ovarian. The results indicated that the inhibition of the NF-*κ*B signaling pathway may develop the antipyroptosis effect of Mox.

Toll-like receptors (TLRs) are conserved pattern-recognition receptors (PRRs), which are activated by a variety of pathogen-associated molecular patterns (PAMPs) [27]. Toll-like receptor 4 (TLR4) is a member of the TLR family which is activated by lipopolysaccharide. TLR4 signaling pathway plays an important role in the innate immune response and is responsible for inflammatory responses. The activation of TLR4 of the cell surface is followed by the activation of myeloid differentiation primary response gene 88 (MyD88), which is a downstream adaptor molecule of TLR4. Activated MyD88 engages the phosphorylation of the I*κ*B complex, followed by the activation of NF-*κ*B [28]. The present study showed that Mox decreased NLRP3, pro-IL-1*β*, and pro-IL-18 through inhibiting the TLR4/MyD88/NF-*κ*B signaling pathway.

## 5. Conclusion

In conclusion, the present study showed that moxibustion had a therapeutic effect on CP-induced premature ovarian failure in rats. The underlying mechanism might be partially attributed to the inhibition of ovarian pyroptosis. On the one hand, Mox directly inhibited TXNIP/NLRP3/caspase-1 signaling-induced pyroptosis, and on the other hand, it indirectly decreased NLRP3, pro-IL-1*β*, and pro-IL-18 through inhibiting TLR4/MyD88/NF-*κ*B signaling. Our results show that inhibiting pyroptosis or giving Mox might be a new treatment for POF.

## Data Availability

The datasets generated during the current study are available from the corresponding author upon reasonable request.

## Supplementary Materials

Supplementary Materials Table S1: critical chemicals and antibodies.
