# Supplementary material for: Moxibustion against Cyclophosphamide-Induced Premature Ovarian Failure in Rats through Inhibiting NLRP3-/Caspase-1-/GSDMD-Dependent Pyroptosis
Source: Evid Based Complement Alternat Med. 2021 Feb 4;2021:8874757. doi: 10.1155/2021/8874757 (PMC7878072; doi:10.1155/2021/8874757)
Supplement: Supplementary Materials — Table S1: critical chemicals and antibodies. [file 8874757.f1.docx]

**Table S1. Critical chemicals and Antibodies.**

| **Reagents** | **Source** | **Identifier** |
| --- | --- | --- |
| Cyclophosphamide | Jiangsu Sheng Di Pharmaceutical | H32020857 |
| Rat IL-1β ELISA Kit | Elabscience | E-EL-R0012 |
| Rat IL-18 ELISA Kit | Elabscience | E-EL-R0567 |
| Rat FSH ELISA Kit | Elabscience | E-EL-R0391c |
| Rat E2 ELISA Kit | Elabscience | E-EL-0152c |
| Rat LH ELISA Kit | Elabscience | E-EL-R3006 |
| Hematoxylin and Eosin Staining Kit | Beyotime | C0105 |
| IHC staining kit | BOSTER | SA1050 |
| DAB staining kit | BOSTER | AR1026 |
| RIPA Lysis Buffer | Beyotime | P0013B |
| Protease and phosphatase inhibitor | Beyotime | P1048 |
| PMSF | Beyotime | ST506 |
| BCA Protein Assay Kit | Beyotime | P0010 |
| SDS-PAGE Sample Loading Buffe | Beyotime | P0015L |
| Rabbit monoclonal anti-TXNIP | Cell Signaling Technology | #14715 |
| Rabbit monoclonal anti-MyD88 | Cell Signaling Technology | #4283 |
| Rabbit monoclonal anti-NF-κB p65 | Cell Signaling Technology | #8242 |
| Rabbit monoclonal anti-ASC | Cell Signaling Technology | #67824 |
| Rabbit monoclonal anti-Cleaved-caspase-1 | Cell Signaling Technology | #89332 |
| Rabbit polyclonal anti-Gasdermin D | Cell Signaling Technology | #93709 |
| Mouse monoclonal anti-TLR4 | Santa cruz | sc-293072 |
| Rabbit polyclonal anti-p-NF-κB p65 | Abcam | ab86299 |
| Rabbit polyclonal anti-NLRP3 | Abcam | ab214185 |
| Rabbit polyclonal anti-IL-1β | Abcam | ab9722 |
| Rabbit polyclonal anti-IL-18 | Abcam | ab191860 |
| Rabbit monoclonal anti-GAPDH | Cell Signaling Technology | #2118 |
| HRP-labeled Goat Anti-Rabbit IgG(H+L) | Beyotime | A0208 |
| HRP-labeled Goat Anti-Mouse IgG(H+L) | Beyotime | A0216 |
| Enhanced Chemiluminescent Kit | Thermo Fisher | 34580 |
